# Myocardial Work Assessment in Patients after Coronary Artery Bypass Grafting during Cardiac Rehabilitation

**DOI:** 10.3390/jcm12247540

**Published:** 2023-12-06

**Authors:** Francesco Perone, Roberta Ancona, Fausto di Stasio, Vito La Gambina, Salvatore Comenale Pinto

**Affiliations:** 1 Cardiac Rehabilitation Unit, Rehabilitation Clinic “Villa delle Magnolie”, 81020 Castel Morrone, Italy; faudis80@gmail.com (F.d.S.); vilagamb@tin.it (V.L.G.); salvcom11@gmail.com (S.C.P.); 2 Pediatric Cardiology Unit and ICCU, A.O.R.N. dei Colli, Monaldi Hospital, “L.Vanvitelli” University, 80131 Naples, Italy; roby.ancona@gmail.com

**Keywords:** myocardial work, cardiac rehabilitation, coronary artery bypass grafting, coronary artery disease, echocardiography, global longitudinal strain, six-minute walk test, speckle-tracking echocardiography

## Abstract

Background: Myocardial work is an innovative echocardiographic tool to assess left ventricular performance. Emerging data have shown the added value of this method for evaluating cardiac function compared to traditional echocardiographic parameters and global longitudinal strain. However, few studies are present in the literature about the role of myocardial work during cardiac rehabilitation. Our aim was to assess the impact of a rehabilitation program on myocardial work indices in patients with preserved left ventricular ejection fraction and after coronary artery bypass grafting. In addition, we assessed the correlation between baseline myocardial work indices and their change after cardiac rehabilitation, establishing an optimal cut-off value to predict the improvement. Methods: An observational, single-center, and prospective study was conducted. We enrolled patients referred to cardiac rehabilitation after coronary artery bypass grafting and with preserved left ventricular ejection fraction. Before and after the cardiac rehabilitation program, a comprehensive patient assessment was performed, including traditional transthoracic echocardiography, myocardial work analysis, and a six-minute walk test. Results: Eighty-four patients were enrolled; the mean age was 67.96 (±7.42) years and 78.6% were male. The left ventricular ejection fraction was preserved in all patients, and the global longitudinal strain was −16.18 ± 2.55%, the global work index was 1588.56 ± 345 mmHg%, the global constructive work was 1771.27 ± 366.36 mmHg%, the global wasted work was 105.8 ± 72.02 mmHg%, and the global work efficiency was 92.63 ± 3.9% at baseline. After the cardiac rehabilitation program, the global work index, the global constructive work, and the six-minute walk test improved significantly (1588.56 ± 345 vs. 1960.2 ± 377.03 mmHg%, *p*-value < 0.001; 1771.27 ± 366.36 vs. 2172.01 ± 418.73 mmHg%, *p*-value < 0.001; 70.71 ± 40.2 vs. 437.5 ± 108.70 m, *p*-value < 0.001, respectively). Conclusions: Myocardial work indices, specifically global work index and global constructive work, improve after cardiac rehabilitation program in patients undergoing coronary artery bypass grafting with preserved left ventricular ejection fraction.

## 1. Introduction

Noninvasive myocardial work (MW) is an emerging echocardiographic tool to evaluate left ventricular (LV) performance [1]. Currently, LV ejection fraction and global longitudinal strain (GLS) are the recommended methods for assessing LV global systolic function, but they have several limitations. Indeed, these modalities are LV load-dependent and do not provide information on metabolic demand [2]. MW overcomes these limitations and measures LV pressure–strain loop area noninvasively [3]. Specifically, the loop area is generated by measuring LV GLS using speckle-tracking echocardiography, and peak LV pressure is estimated using brachial artery cuff pressure. MW assessment integrates LV deformation and afterload, providing information on ventricular efficiency [4].

Cardiac rehabilitation is a comprehensive intervention performed by a multidisciplinary team with the aim of defining a specific and tailored program, including patient assessment, risk factor management, psychosocial intervention, nutritional advice, physical activity counseling, and the prescription of exercise training [5]. This intervention is associated with a significant improvement in prognosis, especially in individuals after acute coronary syndrome (ACS) and after coronary artery bypass grafting (CABG). In these categories of patients, cardiac rehabilitation program reduces hospitalization, myocardial infarction, cardiovascular and all-cause mortality and improves functional capacity [6,7]. The European Society of Cardiology Guidelines recommend cardiac rehabilitation in Class IA, highlighting the key role in the pathway, management, and outcome of patients after CABG or percutaneous coronary intervention for ACS or with chronic coronary syndrome [8,9].

Interest in and implementation of advanced echocardiography in cardiac rehabilitation is growing. Compared to traditional parameters, speckle-tracking echocardiography guarantees repeatability and objective quantification due to low inter- and intra-observer variability [10].

GLS allows the detection of subclinical LV dysfunction in the presence of a preserved LV ejection fraction, adding more accurate and valuable information on systolic function [11,12]. Several studies documented the positive effect of exercise training on GLS, specifically in patients with arterial hypertension [13,14], recent myocardial infarction [15], or recent ST-segment elevation myocardial infarction (STEMI) and LV ejection fraction > 45% [16]. However, other studies showed conflicting data on the improvement in GLS due to the training program [17,18], as well as in oncological patients undergoing cardio-oncology rehabilitation, suggesting the need for further investigations [19]. On the other hand, noninvasive MW has been poorly studied in the field of cardiac rehabilitation. D’Andrea et al. [20] studied 75 patients with recent ACS to assess the effect of high-intensity interval training (HIIT), compared to moderate-intensity continuous training (MICT), on advanced echocardiographic parameters. They found in patients rehabilitated with HIIT protocol an increased MW efficiency (91.1 ± 3.3 vs. 87.4 ± 4.1, *p*-value < 0.01) and reduced myocardial waste work (9.9 ± 4.4 vs. 12.6 ± 3.3, *p*-value < 0.01). Instead, regarding the role of isometric exercise training, O’Driscoll et al. [14] studied 24 unmedicated hypertensive patients and the effect of this training on LV mechanics and global MW indices. The authors documented a significant improvement in global work efficiency (GWE) (2.8  ±  2%, *p*-value  <  0.001) and a reduction in global wasted work (GWW) (− 42.5  ±  30 mmHg%, *p*-value  <  0.001) after 4 weeks of isometric exercise training. 

Noninvasive MW evaluates myocardial systolic performance by overcoming important limitations of LV ejection fraction and GLS measurement. This parameter could provide more accurate information regarding the effect of cardiac rehabilitation programs on systolic function in patients with cardiovascular disease. The first studies published in the literature on exercise training demonstrate its precious added value and reliability. However, further scientific research is needed to confirm these results and allow the use of this new echocardiographic index routinely in clinical practice. Of note, MW has never been studied in patients undergoing CABG with preserved LV ejection fraction and referred to cardiac rehabilitation. Thus, the aim of our study is to assess (1) the effect of cardiac rehabilitation program on LV MW in patients after CABG and with preserved LV ejection fraction, (2) the potential correlation between baseline MW indices and change after cardiac rehabilitation, and (3) the optimal cut-off values of baseline MW indices to predict the improvement through exercise training.

## 2. Materials and Methods

### 2.1. Study Population 

**Figure 1:**
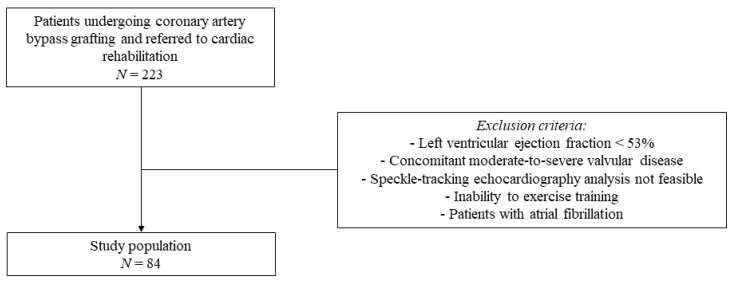
Flow chart of the study population selection.

An observational, single-center, and prospective study was conducted at the Cardiac Rehabilitation Unit of the Rehabilitation Clinic “Villa delle Magnolie”, Castel Morrone, Italy. Patients undergoing CABG and referred to cardiac rehabilitation were enrolled. Exclusion criteria were LV ejection fraction < 53%, concomitant moderate-to-severe valvular disease, speckle-tracking echocardiography analysis not feasible, inability to exercise training, and patients with atrial fibrillation (Figure 1). 

Clinical examination, 12-lead electrocardiogram, blood pressure, routine blood tests, six-minute walk test (6MWT), and basic and advanced transthoracic echocardiography were performed at baseline and after the cardiac rehabilitation program. This study was approved by the Institutional Review Board, and each participant provided informed consensus.

### 2.2. Cardiac Rehabilitation Program, Six-Minute Walk Test, and Borg Scale

Patients enrolled in our study performed comprehensive cardiac rehabilitation with a tailored exercise training program, according to the European Society of Cardiology Guidelines [5,21]. All participants performed aerobic training on a cycle ergometer and/or treadmill with MICT (55–74% maximum heart rate, 40–69% heart rate reserve, and rate of perceived exertion 12–13) and a program lasting up to 4 weeks with 6 sessions/week. The 6MWT was performed before and after the cardiac rehabilitation program. The test was performed according to the latest guidelines [22,23] to evaluate the patient’s functional exercise capacity. Patients were instructed how to perform the test, specifically to walk quickly along a flat surface in 6 min. The total distance walked was calculated in meters. Other monitoring parameters were recorded at rest, during, and end-test as oxyhemoglobin saturation (SpO_2_) and heart rate response. Furthermore, dyspnoea and subjective fatigue were measured before and after the 6MWT using the Borg scale. This scale was used to assess perceived breathlessness and fatigue at baseline and during walking. The rating of the perceived effort is based on a scale from 6 (no exertion at all) to 20 (maximal exertion) [24]. Reasons for test cessation applied to the 6MWT performed before and after cardiac rehabilitation were profound desaturation (SpO_2_ < 80%), intolerable dyspnoea, chest pain, leg cramps and musculoskeletal pain, staggering, and acute hemodynamic complications [23]. 

### 2.3. Echocardiographic Examination and Myocardial Work Analysis

Transthoracic echocardiograms were performed using the Vivid E9 ultrasound system (General Electric Healthcare, Horten, Norway). The same protocol was applied to all participants for performing a comprehensive transthoracic echocardiographic examination, according to the guidelines [25]. For the purpose of this study, the following baseline parameters were assessed and measured: LV end-diastolic diameter, LV end-systolic diameter, LV mass index, indexed LV end-diastolic and end-systolic volumes, two-dimensional LV ejection fraction using the biplane Simpson’s method, three-dimensional LV ejection fraction, stroke volume from the LV outflow tract (LVOT) diameter and LVOT time–velocity integral (TVI), and indexed left atrial volume using the biplane Simpson’s method [2,26,27]. LV GLS by speckle-tracking echocardiography was calculated from the apical four- and two-chamber and long-axis views and expressed in absolute value (%) as the average of peak systolic longitudinal strain values of the 17 segments [2]. Quantification of MW was obtained from noninvasive peak LV pressure using brachial cuff blood pressure, recorded at the time of performing the transthoracic echocardiogram and LV GLS. After indicating the opening and closing time points of the aortic and mitral valve and inserting blood pressure values in the software, an LV pressure–strain loop area was generated [1]. Specifically, chamber quantification, LV GLS, and MW analyses were performed offline using EchoPAC Version 202 software (GE Vingmed Ultrasound, Horten, Norway). The calculated MW indices were global work index (GWI), global constructive work (GCW), GWW, and GWE. Specifically, GWI represents the area within the LV pressure–strain loop and defines the total work from mitral valve closure to opening. This index quantifies the LV work during the whole systole, including isovolumic contraction and isovolumic relaxation time. GCW indicates the positive work performed during isovolumic contraction time and systole (segment shortening) and negative work during isovolumic relaxation time (segment lengthening). GWW represents negative work during isovolumic contraction time and systole (segment lengthening) and positive work during isovolumic relaxation time (segment shortening). GWE is GCW divided by the sum of GCW and GWW, indicating the percentage of constructive work during total work [1,10,28].

### 2.4. Statistical Analysis

Data were presented as mean ± deviation standard with normally distributed variables and as median and interquartile range with non-normally distributed values. Normal distribution was assessed with Kolmogorov–Smirnov test. Instead, categorical data were presented as frequencies and percentages. Changes before and after cardiac rehabilitation program were analyzed for normally distributed variables with paired Student’s *t*-test and Wilcoxon test for non-normally distributed variables. Correlations between variables were assessed using the Pearson’s correlation coefficient (r) and scatter plots. Comparison between groups was performed using Student’s *t*-test or Mann–Whitney U test, as appropriate. Receiver operating characteristic (ROC) analysis was used to establish optimal cut-off values, calculating the area under the curve (AUC) and Youden index (YI). Two-tailed *p*-values < 0.05 were considered statistically significant. Statistical analyses were performed using SPSS version 25.0 (IBM SPSS Statistics for Windows, Armonk, NY, USA).

## 3. Results

Eighty-four patients were prospectively enrolled in our study. Baseline clinical and functional characteristics are summarized in Table 1. All patients underwent CABG and were referred to a cardiac rehabilitation program after 8.5 (±4.9) days. The mean age was 67.96 (±7.42) years, 78.6% were male, and, regarding CV risk factors, the patients suffered predominantly from arterial hypertension (82.1%) and hypercholesterolemia (69%) and almost half from type 2 diabetes mellitus (46.4%) and were past smokers (40.5%). Nearly two-thirds (71.4%) of the participants were patients with chronic coronary syndrome; the rest of the population were individuals with ACS (STEMI 4.8%, non-ST-elevation myocardial infarction 20.2%, and unstable angina 3.6%). Instead, basic and advanced echocardiographic characteristics were provided in Table 2. All patients showed preserved LV ejection fraction using both two-dimensional (60.58 ± 5.26%) and three-dimensional echocardiography (60.49 ± 4.84%). Speckle-tracking-derived GLS was reduced before starting the cardiac rehabilitation program (−16.18 ± 2.55%). MW analysis was performed, and the calculated indices presented the following mean values: GWI 1588.56 ± 345 mmHg%, GCW 1771.27 ± 366.36 mmHg%, GWW 105.8 ± 72.02 mmHg%, and GWE 92.63 ± 3.9%. 

The impact of cardiac rehabilitation on echocardiographic and functional parameters is summarized in Table 3. GLS and MW indices, two out of four, improved after the rehabilitation program. Specifically, GWI and GCW significantly improved (1588.56 ± 345 vs. 1960.2 ± 377.03 mmHg%, *p*-value < 0.001; 1771.27 ± 366.36 vs. 2172.01 ± 418.73 mmHg%, *p*-value < 0.001, respectively) while GWE and GWW remained substantially unchanged (92.63 ± 3.9 vs. 93.15 ± 7.13%, *p*-value 0.196; 105.8 ± 72.02 vs. 117.36 ± 74.92 mmHg%, *p*-value 0.067, respectively). Likewise, 6MWT significantly improved after cardiac rehabilitation (70.71 ± 40.2 vs. 437.5 ± 108.70 m, *p*-value < 0.001). Furthermore, this improvement was quantified by calculating the delta % change (Table 4). The mean improvement after cardiac rehabilitation was 26.32 ± 25.38% for GWI, 25 ± 23.09 % for GCW, 23.52 ± 72.14% for GWW, and 0.65 ± 4.04% for GWE. 

**Figure 2:**
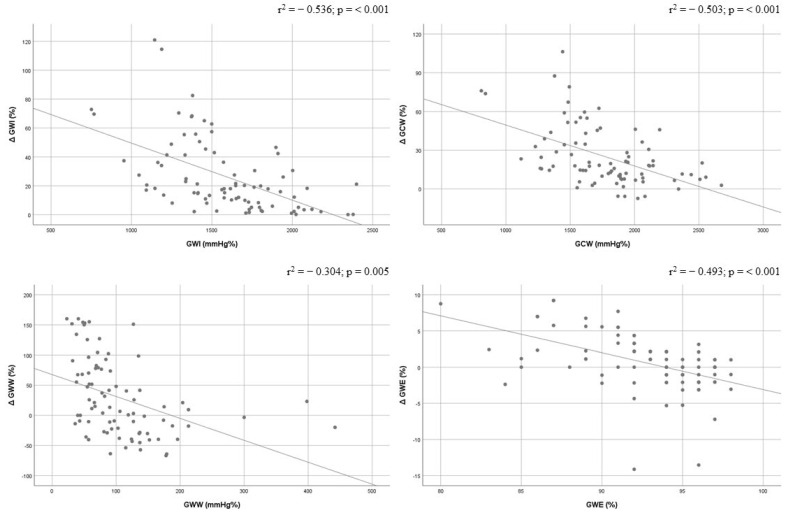
Correlations between baseline values of global work index, global constructive work, global wasted work, and global work efficiency and their delta change (%). Global constructive work, GCW; global work efficiency, GWE; global work index, GWI; global wasted work, GWW.

Correlations between baseline GWI, GCW, GWW, and GWE values and corresponding delta % change were performed. GWI and GCW showed a good correlation (r^2^ = −0.536, *p* < 0.001; and r^2^ = −0.503, *p* < 0.001), and GWW and GWE documented a moderate correlation (r^2^ = −0.304, *p* = 0.005; and r^2^ = −0.493, *p* < 0.001). All baseline MW indices were inversely correlated with the degree of delta % change improvement (Figure 2).

**Figure 3:**
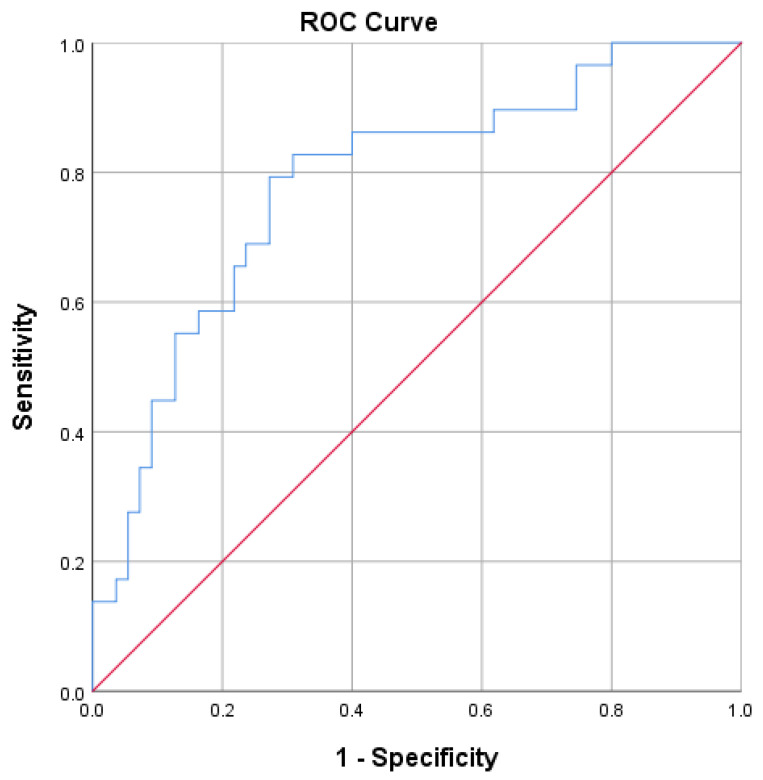
Receiver operator characteristic (ROC) curve for predicting an improvement in GWI ≥ 26.32%.

**Figure 4:**
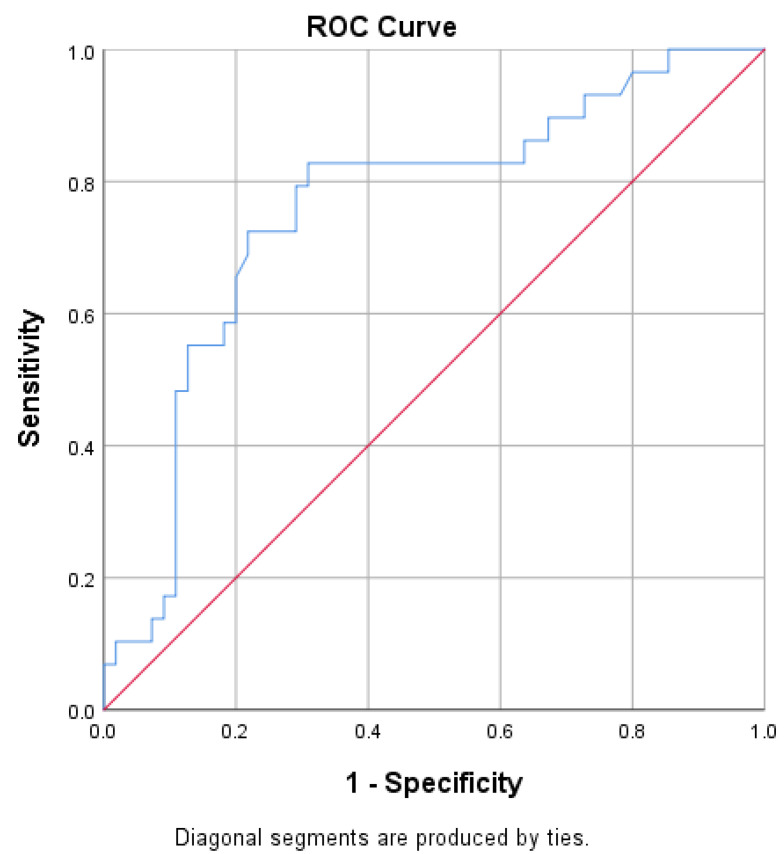
Receiver operator characteristic (ROC) curve for predicting an improvement in GCW ≥ 25%.

ROC curves for predicting delta % change in GWI and GCW were developed (Figure 3 and Figure 4). Optimal GWI cut-off value of 1522.5 mmHg% predicted an improvement ≥ 26.32% with a sensitivity of 79% and specificity of 73% (area 0.784; 95% CI = 0.680–0.888; *p* < 0.001; Youden index 0.520). On the other hand, the optimal GCW cut-off value of 1740.5 mmHg% was associated with an improvement ≥ 25 % with a sensitivity of 83% and specificity of 69% (area 0.759; 95% CI = 0.647–0.870; *p* < 0.001; Youden index 0.518).

## 4. Discussion

Our study shows the following main findings: (1) cardiac rehabilitation improves MW indices (GWI and GCW) after a 4-week program with 6 sessions/week and MICT in patients after CABG with preserved LV ejection fraction; (2) improvement in MW indices was inversely correlated with baseline values; and (3) optimal cut-off values were defined to predict significant improvement after the cardiac rehabilitation program, specifically 1522.5 mmHg% for GWI and 1740.5 mmHg% for GCW.

**Figure 5:**
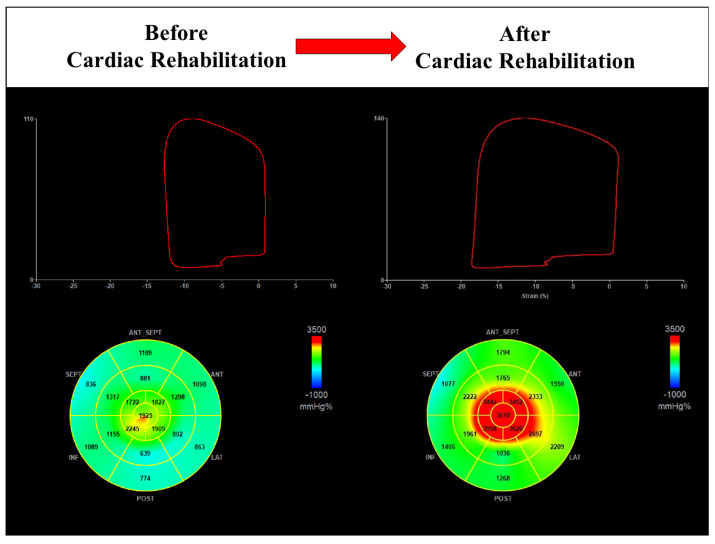
Cardiac rehabilitation improves global work index in a male patient with preserved value at baseline (1301 mmHg% vs. 2394 mmHg%).

Guidelines on cardiac rehabilitation [5] suggest LV systolic function assessment during baseline evaluation before starting the program. However, LV ejection fraction is limited in stratifying patients with preserved systolic function in cardiac rehabilitation and evaluating the impact and benefit of the program. Furthermore, subclinical systolic dysfunction could be undiagnosed or underestimated [29]. Advanced echocardiography could add key information in this setting with speckle-tracking-echocardiography-derived parameters. Currently, studies on the role of GLS [13,14,15,16,17,18,19] in evaluating the effect of exercise training on myocardial function have shown conflicting data. Moreover, the method is affected by various limitations, such as load dependence and lack of information on metabolic demand. MW assessment overcomes these limitations by providing valuable information on LV performance and also detecting subclinical dysfunction [4,28]. In the field of cardiac rehabilitation, very few studies have been published in the literature on the application of this new emerging echocardiographic method and its impact. Our study adds new data on the application of MW in patients with coronary artery disease during cardiac rehabilitation. Furthermore, our findings are the first regarding patients undergoing CABG and with preserved LV ejection fraction. We found an improvement in GWI and GCW after the rehabilitation program and a beneficial effect on cardiac performance (Figure 5). A previous study [20] documented an improvement in deformation indices, such as GLS, left atrial strain, and MW efficiency (91.1 ± 3.3 vs. 87.4 ± 4.1, *p*-value < 0.01), after cardiac rehabilitation in 75 patients with recent ACS. These indices significantly improved after HIIT compared to MICT after 8 weeks of training. As in our study, D’Andrea et al. documented that MW indices were sensible markers of myocardial systolic function in detecting early damage and changes after cardiac rehabilitation. Furthermore, especially in patients with normal LV ejection fraction, these indices are fundamental to quantifying LV remodeling and contractility change. Previous studies [16,30,31,32] have already demonstrated the limitations of LV ejection fraction in diagnosing early deterioration of systolic function and the superiority of speckle-tracking-echocardiography-derived parameters. Our study further highlights the key role of advanced echocardiography in stratifying the patient, demonstrating early changes in systolic function, and evaluating the impact of cardiac rehabilitation not quantifiable with traditional echocardiographic parameters, such as LV ejection fraction.

**Figure 6:**
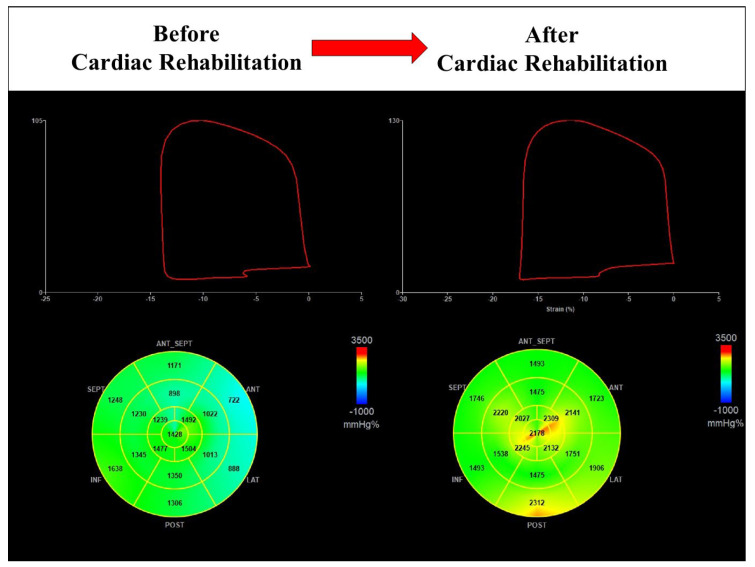
Cardiac rehabilitation improves global work index in a male patient with reduced value at baseline (1244 mmHg% vs. 1908 mmHg%).

As a second finding, our study demonstrated that improvement in MW indices was inversely related to baseline values. Patients with lower values before cardiac rehabilitation showed greater improvement after the program. This result could suggest a different type of training in individuals with high values of MW indices at baseline. In our study, all enrolled participants were referred to a 4-week program with MICT. Therefore, in patients with good cardiac performance before starting the program, HIIT could be prescribed for this specific target of intervention [21]. Furthermore, our study showed that 18% of patients had reduced GWI at baseline (1097 ± 158.17 vs. 1695.42 ± 274.31 mmHg%), but all recovered after the cardiac rehabilitation program (Figure 6). On the other hand, participants showed a significant improvement in functional capacity compared to baseline (70.71 ± 40.2 vs. 437.5 ± 108.70 m, *p* < 0.001). The 6MWT performed before the cardiac rehabilitation program documented a reduced total distance walked that was also due to physical limitations and complications related to cardiac surgery. In our population characterized by individuals with coronary artery disease undergoing CABG and preserved LV ejection fraction, the 4-week cardiac rehabilitation program with MICT was an effective aerobic training modality as it resulted in a significant improvement in cardiac performance and functional capacity. 

As a third finding, we found specific optimal cut-off values to predict the improvement in MW indices in patients after cardiac rehabilitation completion. Specifically, GWI of 1522.5 mmHg% and GCW of 1740.5 mmHg% predicted improvements of ≥26.32% and ≥25%, respectively. These values could be useful in clinical practice to stratify the individuals and define a tailored cardiac rehabilitation program [5,21,33]. GWI and GCW represent the total work performed during LV mechanical systole and the energy required to contribute to cardiac output. Our study proposes MW thresholds to predict exercise training response and to identify patients who could substantially improve cardiac performance with a cardiac rehabilitation program. This is a key point, as cardiac rehabilitation has an extremely positive prognostic effect. Indeed, European Guidelines suggest this intervention in Class IA after acute or chronic coronary syndrome [8,9,34]. After atherosclerotic events and/or coronary revascularization, cardiac rehabilitation reduces hospitalizations, myocardial infarction, and mortality. Rauch et al. [6] found a consistent reduction in total mortality after CABG in the modern era (hazard ratio 0.62, 95% confidence interval 0.54–0.70). Subsequently, Salzwedel et al. [7] confirmed similar data in this setting. After ACS, Anderson et al. [35] documented that cardiac rehabilitation reduced CV mortality by 26% (relative risk 0.74; 95% confidence interval 0.64–0.86) and risk of hospitalization by 18% (relative risk 0.82; 95% confidence interval 0.70–0.96). In individuals with coronary heart disease, Dibben et al. [36] described a significant reduction in myocardial infarction during short- (6–12 months) and long-term (>3 years) follow-up after exercise-based cardiac rehabilitation (risk ratio 0.72, 95% confidence interval 0.55–0.93 and risk ratio 0.67, 95% CI 0.50–0.90, respectively). This intervention in patients with coronary artery disease increases survival by reducing mortality and hospitalizations [34,37]. This consistent data demonstrates the importance of a tailored and specific assessment before starting the program. Indeed, the goal should be to identify the specific program and the patient responding to cardiac rehabilitation and improvement in cardiac function, exercise capacity, and prognosis. Advanced echocardiography and MW analysis should be part of the routine evaluation in the clinical practice for the capacity to assess cardiac performance, early and subclinical myocardial dysfunction, and effective change after therapies and interventions such as cardiac rehabilitation. LV MW adds valuable information on cardiac function and myocardial performance; however, further studies are necessary to confirm emerging data. Multicenter studies with a large population are required to perform this innovative tool in clinical practice. Future research should analyze specific populations, such as patients with coronary artery disease, to better stratify the risk and identify responders and non-responders to cardiac rehabilitation. In this case, the selection of exercise training modality based on MW indices could play a key role. In addition, the prognostic value of MW indices in this setting should be investigated. A specific cut-off value could identify high-risk patients after cardiac rehabilitation who need further interventions to reduce residual CV risk. Compared with previous studies, further research should be performed to confirm the role of all four MW indices in stratifying patients with coronary artery disease during cardiac rehabilitation.

Our work presents several limitations. First, this is a single-center study. Second, exercise intensity prescription was not assessed by cardiopulmonary testing or exercise testing, as suggested by the European Position Papers [5,21]. However, we used necessary and valid alternatives because our patients could not perform exercise testing due to physical limitations and complications after cardiac surgery [38]. Third, speckle-tracking echocardiography is dependent on frame rate and image resolution, but we selected only patients with optimal image quality. Fourth, patients with atrial fibrillation were excluded to avoid technical errors related to cardiac rhythm during advanced echocardiography analysis, introducing a potential selection bias. On the other hand, as a strength, our study is the first to analyze MW in patients referred to cardiac rehabilitation with preserved LV ejection fraction and undergoing CABG. In addition, our study establishes optimal cut-off values of baseline GWI and GCW to predict their significant improvement after a 4-week cardiac rehabilitation program with MICT.

## 5. Conclusions

Cardiac rehabilitation improves MW indices as GWI and GCW in patients with preserved LV ejection fraction and after CABG. The beneficial impact is significant on cardiac performance and functional capacity. Lower values of MW indices were associated with greater improvement after cardiac rehabilitation. Optimal cut-off values of 1522.5 mmHg% for GWI and 1740.5 mmHg% for GCW predicted improvements of ≥26.32% and ≥25%, respectively. Further studies are necessary to include MW analysis in routine assessment during cardiac rehabilitation and to identify high-risk patients and worse long-term outcomes.

## Figures and Tables

**Table 1:** Baseline characteristics of the study population.

Variable	Total (*n* = 84)
Age, years	67.96 ± 7.42
Male sex, *n* (%)	66 (78.6)
BSA, m^2^	1.83 ± 0.17
BMI, kg/m^2^	26.93 (25–29)
Arterial hypertension, *n* (%)	69 (82.1)
Type 2 diabetes mellitus, *n* (%)	39 (46.4)
Hypercholesterolemia, *n* (%)	58 (69)
Hypertriglyceridemia, *n* (%)	5 (6)
Active smoker, *n* (%)	19 (22.6)
Past smoker, *n* (%)	34 (40.5)
Previous myocardial infarction, *n* (%)	18 (21.4)
STEMI, *n* (%)	4 (4.8)
NSTEMI, *n* (%)	17 (20.2)
Unstable angina, *n* (%)	3 (3.6)
Chronic coronary syndrome, *n* (%)	60 (71.4)
Coronary artery bypass graft surgery • One, *n* (%) • Two, *n* (%) • Three, *n* (%) • Four, *n* (%)	15 (17.9) 41 (48.8) 26 (31) 2 (2.4)
Concomitant other cardiac surgery • Mitral valve surgery, *n* (%) • Aortic valve surgery, *n* (%)	3 (3.6) 15 (17.9)
Aspirin, *n* (%)	60 (71.4)
Dual antiplatelet therapy, *n* (%)	24 (28.6)
Anticoagulants, *n* (%)	9 (10.7)
ACE-inhibitors or AT-receptor antagonists, *n* (%)	62 (73.8)
Beta-blockers, *n* (%)	79 (94)
Mineralocorticoid receptor antagonists, *n* (%)	61 (72.6)
Statins, *n* (%)	69 (82.1)
Loop diuretics, *n* (%)	72 (85.7)
Six-minute walk test, meters	70.71 ± 40.2

ACE, angiotensin-converting enzyme; AT, angiotensin; BMI, body mass index; BSA, body surface area; NSTEMI, non-ST-elevation myocardial infarction; STEMI, ST-elevation myocardial infarction.

**Table 2:** Baseline echocardiographic assessment.

Variable	Total (*n* = 84)
Heart rate (bpm)	78.8 ± 13.13
Systolic blood pressure (mmHg)	118.45 ± 17.58
Diastolic blood pressure (mmHg)	67.8 ± 6.78
LVEDD (cm)	4.28 ± 0.6
LVESD (cm)	2.56 ± 0.55
LV mass index (g/m^2^)	98.29 ± 2.6
LVEDV index (mL/m^2^)	28.08 (23.18–37.37)
LVESV index (mL/m^2^)	11.55 (8.77–15.9)
2D LV ejection fraction (%)	60.58 ± 5.26
3D LV ejection fraction (%)	60.49 ± 4.84
Global longitudinal strain (%)	−16.18 ± 2.55
Global work index (mmHg%)	1588.56 ± 345
Global constructive work (mmHg%)	1771.27 ± 366.36
Global wasted work (mmHg%)	105.8 ± 72.02
Global work efficiency (%)	92.63 ± 3.9
LAV index (mL/m^2^)	31.54 (27.59–38.38)
Stroke volume (mL)	60.05 ± 19.44

2D, two-dimensional; 3D, three-dimensional; LAV, left atrial volume; LV, left ventricular; LVEDD, LV end-diastolic diameter; LVESD, LV end-systolic diameter; LVEDV, LV end-diastolic volume; LVESV, LV end-systolic volume.

**Table 3:** Echocardiographic and functional parameters before and after cardiac rehabilitation.

Parameter	Before Cardiac Rehabilitation	After Cardiac Rehabilitation	*p*-Value
2D left ventricle ejection fraction (%)	60.58 ± 5.26	62 ± 5.13	<0.001
3D left ventricle ejection fraction (%)	60.49 ± 4.49	62.17 ± 4.67	0.001
Global longitudinal strain (%)	−16.17 ± 2.55	−18.27 ± 2.39	<0.001
Global work index (mmHg%)	1588.56 ± 345	1960.2 ± 377.03	<0.001
Global constructive work (mmHg%)	1771.27 ± 366.36	2172.01 ± 418.73	<0.001
Global wasted work (mmHg%)	105.8 ± 72.02	117.36 ± 74.92	0.067
Global work efficiency (%)	92.63 ± 3.9	93.15 ± 7.13	0.196
Six-minute walk test (meters)	70.71 ± 40.2	437.5 ± 108.70	<0.001

2D, two-dimensional; 3D, three-dimensional.

**Table 4:** Myocardial work indices delta change (%).

Parameter	Δ%
Global work index (%)	26.32 ± 25.38
Global constructive work (%)	25 ± 23.09
Global wasted work (%)	23.52 ± 72.14
Global work efficiency (%)	0.65 ± 4.04

## Data Availability

The data presented in this study are available on request from the corresponding author.
